# Membrane Bridging and Hemifusion by Denaturated Munc18

**DOI:** 10.1371/journal.pone.0022012

**Published:** 2011-07-06

**Authors:** Yi Xu, Alpay B. Seven, Lijing Su, Qiu-Xing Jiang, Josep Rizo

**Affiliations:** 1 Department of Biochemistry, University of Texas Southwestern Medical Center, Dallas, Texas, United States of America; 2 Department of Pharmacology, University of Texas Southwestern Medical Center, Dallas, Texas, United States of America; 3 Department of Cell Biology, University of Texas Southwestern Medical Center, Dallas, Texas, United States of America; Griffith University, Australia

## Abstract

Neuronal Munc18-1 and members of the Sec1/Munc18 (SM) protein family play a critical function(s) in intracellular membrane fusion together with SNARE proteins, but the mechanism of action of SM proteins remains highly enigmatic. During experiments designed to address this question employing a 7-nitrobenz-2-oxa-1,3-diazole (NBD) fluorescence de-quenching assay that is widely used to study lipid mixing between reconstituted proteoliposomes, we observed that Munc18-1 from squid (sMunc18-1) was able to increase the apparent NBD fluorescence emission intensity even in the absence of SNARE proteins. Fluorescence emission scans and dynamic light scattering experiments show that this phenomenon arises at least in part from increased light scattering due to sMunc18-1-induced liposome clustering. Nuclear magnetic resonance and circular dichroism data suggest that, although native sMunc18-1 does not bind significantly to lipids, sMunc18-1 denaturation at 37°C leads to insertion into membranes. The liposome clustering activity of sMunc18-1 can thus be attributed to its ability to bridge two membranes upon (perhaps partial) denaturation; correspondingly, this activity is hindered by addition of glycerol. Cryo-electron microscopy shows that liposome clusters induced by sMunc18-1 include extended interfaces where the bilayers of two liposomes come into very close proximity, and clear hemifusion diaphragms. Although the physiological relevance of our results is uncertain, they emphasize the necessity of complementing fluorescence de-quenching assays with alternative experiments in studies of membrane fusion, as well as the importance of considering the potential effects of protein denaturation. In addition, our data suggest a novel mechanism of membrane hemifusion induced by amphipathic macromolecules that does not involve formation of a stalk intermediate.

## Introduction

Membrane fusion is critical for an immense variety of biological processes, including entry of enveloped viruses into host cells, egg fertilization by sperm, all steps of the secretory and endocytic pathways, and many processes that depend on these pathways. All forms of physiological membrane fusion are believed to share a common basic mechanism involving the formation of a so-called stalk intermediate where the proximal leaflets of two apposed membranes have merged; after stalk formation, the distal leaflets normally fuse, yielding a fusion pore, but can also expand and form a hemifusion diaphragm [1]–[4]. This mechanism appears to prevail regardless of the proteins involved, which can share some common structural features such as coiled-coils, but can also be structurally diverse [5]–[7].

Most types of intracellular membrane fusion are governed by protein machineries that contain members of several conserved protein families, including N-ethyl maleimide sensitive factor (NSF), soluble NSF attachment proteins (SNAPs) [6], SNAP receptors (SNAREs) [8], Sec1/Munc18 (SM) proteins [9], Rab GTPases [10] and tethering factors [11], [12]. Among these proteins, the SNAREs are particularly crucial for membrane fusion, forming tight four-helix bundles called SNARE complexes that bridge the two membranes and bring them into close proximity [13]–[15]. While reconstitution experiments led to the proposal that SNARE complexes constitute a ‘minimal’ membrane fusion machinery [16], and a single SNARE complex may in fact be sufficient for membrane fusion [17], this minimal model is under debate [6]–[8] and very diverse results have been obtained in reconstitution studies with SNAREs alone depending on the conditions used (e.g. [18]–[24]). Moreover, this minimal model does not explain why intracellular membrane fusion in vivo depends critically on other proteins in addition to SNAREs, most notably on SM proteins.

The importance of SM proteins has been demonstrated by the severe or complete blocks in membrane fusion observed in their absence [9], [25], [26], but the function(s) of SM proteins remains enigmatic. SM proteins interact with SNAREs in diverse modes, as has been well illustrated by studies of the neuronal machinery involved in neurotransmitter release, which includes the SM protein Munc18-1 and the SNAREs syntaxin-1, SNAP-25 and synaptobrevin [7], [26]. Thus, Munc18-1 binds to syntaxin-1 folded into a so-called closed conformation that hinders SNARE complex assembly [27]–[29], and to SNARE complexes formed by syntaxin-1, SNAP-25 and synaptobrevin [30], [31]. Both of these interactions involve the N-terminal H_abc_ domain [32] of syntaxin-1 and a preceding sequence at its very N-terminus [30], [31], [33]–[35]. In addition, Munc18-1 binds to the SNARE four-helix bundle [31], [36]. It is still unclear which of these interactions are universally conserved in all types of intracellular membrane traffic and how they are coordinated during the steps that lead to membrane fusion, but it appears that all SM proteins bind to SNARE complexes [7], [26], as originally observed for yeast Sec1p [37], and that this binding underlies how SM proteins and SNAREs cooperate in membrane fusion. One model of how such cooperation arises predicts that binding of the SM protein to the SNARE four-helix is fundamental to enable efficient application of leverage by the SNARE complex on the membranes to induce fusion [3], whereas another model postulates that SM-protein binding causes fusion by changing the membrane curvature preference of an intermediate formed after the SNARE complex has induced hemifusion [9].

Regardless of which model is correct, the overall notion that SM proteins play a key role in membrane fusion was supported by the findings that Munc18-1 substantially enhances lipid mixing between SNARE-containing proteoliposomes in reconstitution assays [31], [38] and is essential for lipid mixing between small and giant vesicles [39]. However, contradictory results have been obtained regarding the sequence requirements for the stimulation of lipid mixing [31], [40], [41], and the mechanism of SM protein/SNARE coupling in membrane fusion remains unknown. During studies directed at addressing this question employing a widely used 7-nitrobenz-2-oxa-1,3-diazole (NBD) fluorescence de-quenching assay [16] in combination with cryo-electron microscopy (cryo-EM) and other biophysical techniques, we have made the unexpected observation that Munc18-1 from squid (sMunc18-1) can induce clustering of liposomes and hemifusion by itself, in the absence of SNAREs. This activity appears to arise from denaturation of sMunc18-1 and is hindered in the presence of glycerol. While the physiological relevance of this activity of sMunc18-1 is unclear, our data emphasize that the results of the NBD fluorescence de-quenching assays need to be interpreted with caution. Thus, increases in NBD fluorescence intensity in these assays are commonly interpreted as a prove of lipid mixing and even as a prove of membrane fusion, but our data show that such increases can arise at least in part from light scattering caused by liposome clustering. In addition, our results illustrate how denatured proteins can strongly alter membranes and suggest a novel mechanism of membrane hemifusion that does not involve a stalk intermediate but rather the formation of extensive membrane-membrane interfaces bridged by amphipathic macromolecules.

## Results

### Liposome clustering induced by sMunc18-1

At physiological pH and ionic strength, rat Munc18-1 (rMunc18-1) has a tendency to precipitate at concentrations above 20 µM, whereas sMunc18-1 can be readily concentrated above 100 µM without precipitation. Since sMunc18-1 still binds to mammalian syntaxin-1 and SNARE complexes with high affinity [36], we have started to perform some experiments with sMunc18-1 in our efforts to study Munc18-1 function. To investigate whether sMunc18-1 can stimulate lipid mixing between liposomes containing synaptobrevin (v-SNARE liposomes) and liposomes containing syntaxin-1/SNAP-25 (t-SNARE liposomes), as described for rMunc18-1 [31], [38], we prepared v-SNARE liposomes containing NBD-labeled lipids and lissamine rhodamine B (Rho)-labeled lipids. In these liposomes, the NBD fluorescence emission is quenched by fluorescence resonance energy transfer (FRET) to the Rho-acceptor groups. Lipid mixing with unlabeled t-SNARE liposomes leads to dilution of the labeled lipids and dequenching of the NBD fluorescence [16].

**Figure 1:**
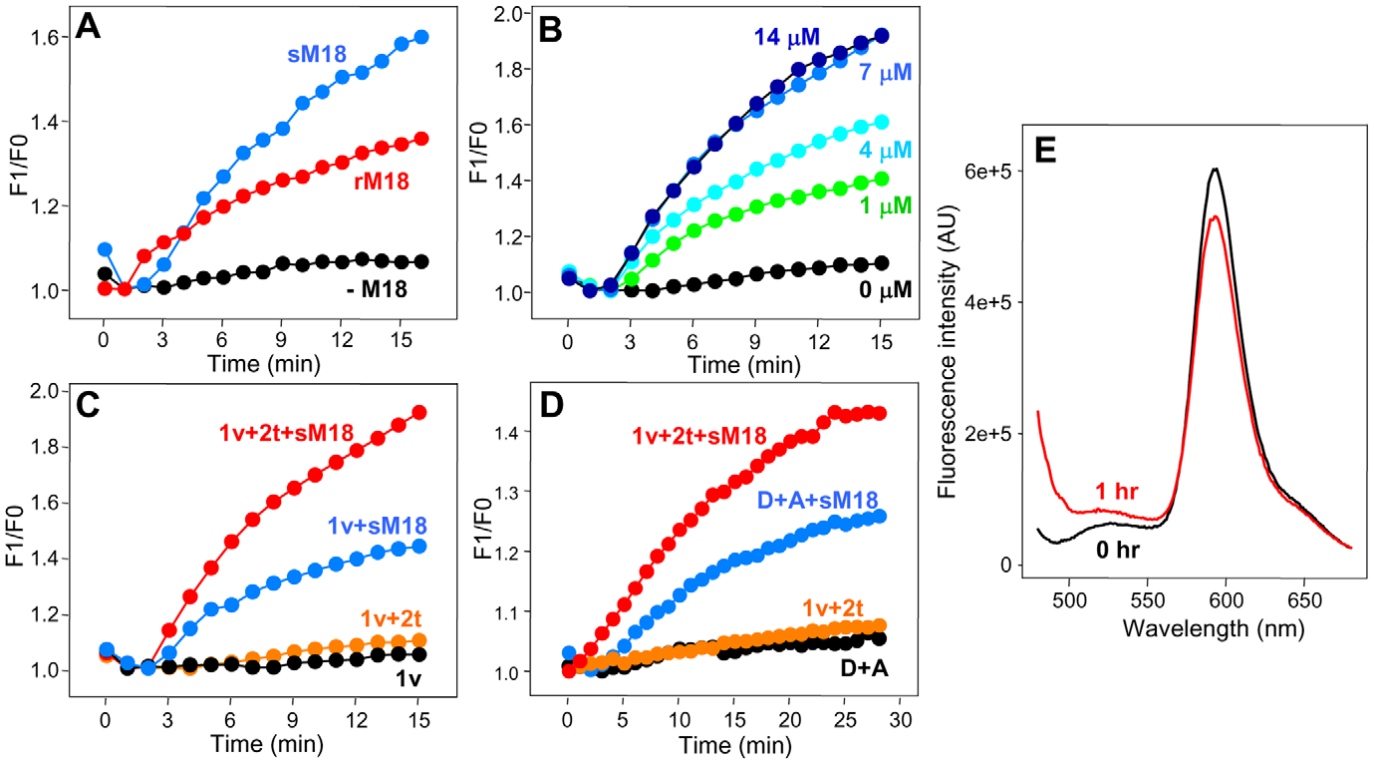
sMunc18-1 can induce SNARE-independent increases in the apparent NBD fluorescence intensity in lipid mixing assays. (**A–C**) Plots of the ratio between observed fluorescence intensity (F1) and the initial fluorescence intensity (F0) during assays intended to monitor lipid mixing through NBD fluorescence de-quenching. The experiments were performed using proteoliposomes containing synaptobrevin (v) or co-expressed syntaxin-1/SNAP-25 (t) reconstituted using the standard method with a 1∶1000 protein-to-lipid ratio and a lipid composition consisting of POPC∶POPE∶DOPS∶PI∶cholesterol 50∶20∶10∶10∶10 (molar ratio). In the v-SNARE liposomes, 3% of POPC was replaced with 1.5% NBD-PE and 1.5% Rho-PE. In (**A**), v-SNARE liposomes (50 µM lipids) and t-SNARE liposomes (50 µM lipids) where mixed in the absence of Munc18-1 (black circles), or in the presence of 4 µM rMunc18-1 (red circles) or 4 µM sMunc18-1 (blue circles). In (**B**), v-SNARE liposomes (50 µM lipids) and t-SNARE liposomes (100 µM lipids) where mixed in the presence of the indicated concentrations of sMunc18-1. In (**C**), reactions contained v-SNARE liposomes (50 µM lipids) without (black circles) or with 7 µM sMunc18-1 (blue circles), or v-SNARE liposomes (50 µM lipids) and t-SNARE liposomes (100 µM lipids) without (orange circles) or with 7 µM sMunc18-1 (red circles). (**D**) Lipid mixing assays performed similarly to (A–C) but using protein-free donor liposomes (D) (50 µM lipids) and protein free acceptor liposomes (A) (100 µM lipids) in the absence (black circles) or presence of 7 µM sMunc18-1 (blue circles), or v-SNARE liposomes (50 µM lipids) and t-SNARE liposomes (100 µM lipids) in the absence (orange circles) or presence of 7 µM sMunc18-1 (red circles). For these experiments, the proteoliposomes were prepared with the direct method, using a protein-to-lipid ratio of 1∶1000 and a lipid composition consisting of POPC∶DOPS 85∶15 (molar ratio) (3% of POPC was replaced with 1.5% NBD-PE and 1.5% Rho-PE for donor liposomes and v-SNARE liposomes). All experiments in (**A**–**D**) were performed at 37°C monitoring the fluorescence emission intensity at 533 nm (excitation at 460 nm). (**E**) Fluorescence emission spectra of the sample used to perform the experiments with D+A liposomes and 7 µM sMunc18-1 of panel (**D**) (blue circles), at the start of the reaction (black trace) and after 1 hr incubation (red trace).

Lipid mixing experiments performed in the presence of sMunc18-1 or rMunc18-1 led to stronger increases in NBD fluorescence over time than those observed with v-SNARE and t-SNARE liposomes alone, but we consistently observed that sMunc18-1 caused larger enhancements than rMunc18-1 (e.g. Fig. 1A). A titration showed that increasing concentrations of sMunc18-1 yielded progressively stronger increases in NBD fluorescence and that the stimulatory effect saturates at 7 µM sMunc18-1 (Fig. 1B), suggesting that the effect is specific. However, and surprisingly, control experiments with only v-SNARE liposomes and sMunc18-1 still revealed a substantial increase in NBD fluorescence intensity (Fig. 1C, blue circles). This effect cannot be due to lipid mixing, since there were no t-SNARE liposomes in these experiments, and was caused by sMunc18-1, since practically no increase in NBD fluorescence was observed in experiments with v-SNARE liposomes in the absence of sMunc18-1 (Fig. 1C, black circles).

The experiments described above were performed with proteoliposomes prepared by the so-called standard reconstitution method, which involves co-solubilization of lipids and membrane proteins with detergent, followed by detergent removal. We also performed NBD fluorescence de-quenching assays with v-SNARE and t-SNARE proteoliposomes prepared by the so-called direct method, which involves detergent-assisted insertion of membrane proteins into preformed liposomes [23]. The presence of sMunc18-1 again led to larger increases in NBD fluorescence, compared to experiments performed with v-SNARE and t-SNARE liposomes (compare red circles and orange circles in Fig. 1D). Intriguingly, sMunc18-1 also caused considerable increases in NBD fluorescence in control experiments performed in the complete absence of SNAREs, i.e. using donor plain liposomes containing the same mixture of NBD- and Rho-labeled lipids as the v-SNARE liposomes, but without synaptobrevin, and acceptor plain liposomes containing no fluorescent lipids and no t-SNAREs (Fig. 1D blue circles). Comparison of fluorescence emission scans acquired immediately after mixing donor and acceptor liposomes in the presence of sMunc18-1 and after 1 hour of incubation revealed wavelength-dependent enhancements in fluorescence intensity that were much stronger at the shortest wavelengths and decayed steeply with increasing wavelength (Fig. 1E). This observation strongly suggests that much of the enhancement in fluorescence intensity at the wavelengths characteristic of NBD arises from increased light scattering rather than from actual NBD fluorescence de-quenching.

**Figure 2:**
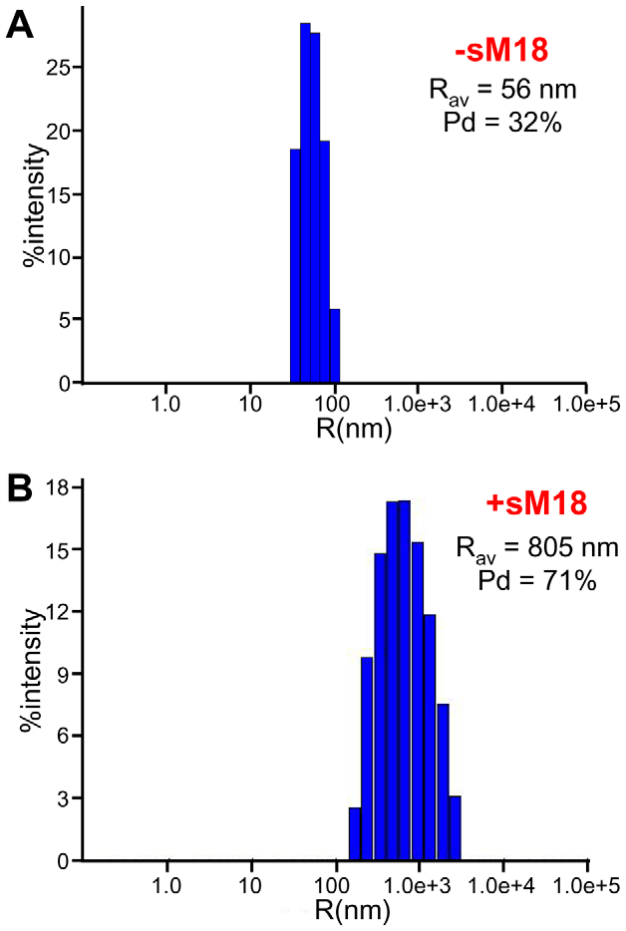
Liposome clustering activity of sMunc18-1. (**A,B**) DLS measurements of particle size in samples containing protein-free liposomes (POPC∶DOPS 85∶15 molar ratio; 30 µM lipids) in the absence (**A**) or presence (**B**) of 4 µM sMunc18-1 after 10 min incubation at 37°C. The average radius (R_av_) and polydispersity (Pd) are indicated.

The increased light scattering could in principle arise from increased liposome size resulting from fusion, but the modest nature of the NBD fluorescence intensity increase and of the decrease in Rho fluorescence intensity that we observed (Fig. 1E) shows that lipid mixing and fusion could only occur to a small extent in these experiments. Hence, we hypothesized that the increased scattering arises in large part from liposome clustering induced by sMunc18-1. To test this hypothesis, we prepared plain liposomes with nominal radii of 50 nm and analyzed the particle size before and after addition of sMunc18-1 by dynamic light scattering (DLS). Analysis of the plain liposomes revealed the expected particle size (Fig. 2A, Table 1), which remained stable over time. However, addition of sM18-1 at 37°C, the temperature used for the NBD fluorescence de-quenching assays, led to dramatic increases in particle size, reaching average particle radii (R_av_) of more than 500 nm after 10 min (Fig. 2B, Table 1). Clearly, such massive increases in particle size cannot result from massive liposome fusion, given the results of the fluorescence emission scans (Fig. 1E). Hence, the most likely explanation for these results is that sMunc18-1 induces the formation of large liposome clusters.

**Table 1:** Liposome clustering activity of sMunc18-1 under different conditions measured by DLS.^a^

	Temperature(°C)	Time	R_av_ (nm)
Liposomes	25/37		55–80
Liposomes+4 µM sMunc18-1	37	5 min	151
Liposomes+4 µM sMunc18-1	37	10 min	>500
Liposomes+4 µM sMunc18-1+1M NaCl	37	10 min	55
Liposomes+4 µM sMunc18-1, after 10 min added 1 M NaCl	37		>500
Liposomes+4 µM sMunc18-1+4 µM Syx	37	10 min	139
Liposomes+4 µM sMunc18-1, after 10 min added 20 µM Syx	37		>500
Liposomes+4 µM sMunc18-1	25	2 hr	60
Liposomes+4 µM sMunc18-1	25	O/N	131

a DLS measurements of particle size in samples containing protein-free liposomes (POPC∶DOPS 85∶15 molar ratio; 30 µM lipids) and the reagents indicated at the left column. The temperature, incubation time and average radius measured (R_av_) are indicated in the other columns.

**Figure 3:**
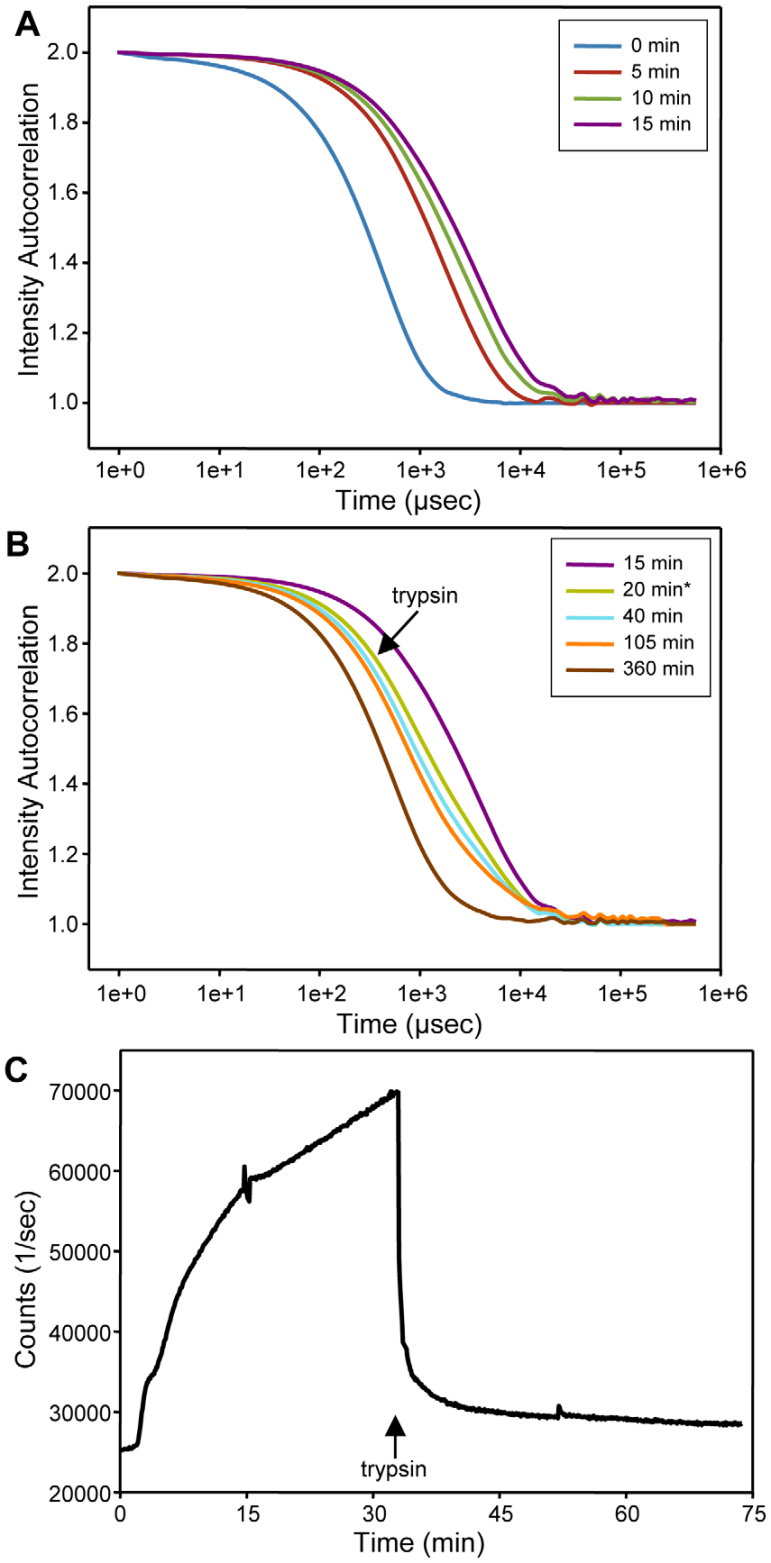
Liposome clustering induced by sMunc18-1 is reversed by trypsinolysis. (**A,B**) Autocorrelation functions obtained by DLS at different time points after mixing protein-free liposomes (POPC∶DOPS 85∶15 molar ratio; 100 µM lipids) with 7 µM sMunc18-1 (**A**), and after adding 0.7 µM trypsin at the 20 min time point (**B**). The insets indicate the color codes for the times at which the data were acquired. Note that the starting point in panel (**B**) is the same curve as the last point of panel (**A**), and that the times indicated in panel (**B**) refer to the beginning of the clustering reaction, rather than the point of trypsin addition. (**C**) Apparent fluorescence signal intensity at 375 nm (excitation at 350 nm) observed as a function of time after mixing protein-free liposomes (POPC∶DOPS 85∶15 molar ratio; 100 µM lipids) with 7 µM sMunc18-1. Trypsin (0.7 µM) was added to the reaction at 33 min. All the experiments in panels (**A–C**) were performed at 37°C.

To test this hypothesis and investigate whether the putative liposome clustering is reversible, we acquired DLS data as a function of time after mixing plain liposomes with sMunc18-1, and added trypsin after 20 min of incubation. Figure 3A shows how the autocorrelation function obtained by DLS gradually shifted to the right (reflecting the formation of larger particles) with increasing time, and Figure 3B shows that the autocorrelation function shifted back to the left upon addition of trypsin (we present these plots because they allow easier visualization of the time-dependence of the DLS data than the radius bar charts shown in Figure 2). In multiple experiments performed, the average particle size consistently increased from 50–60 nm at the start of the reaction to more than 500 nm after 15 min, and it consistently decreased back to the original size for much of the lipid mass (≥90%) after trypsinolysis. However, a small amount of the lipid mass (≤10%) retained a large particle size. We also performed parallel experiments where light scattering as a function of time was monitored with a fluorimeter, measuring the apparent fluorescence signal at 375 nm with excitation at 350 nm. Addition of sMunc18-1 to the liposomes caused a gradual increase in signal with time, while addition of trypsin led to a fast decrease in signal that reach a plateau close to the original value (Fig. 3C).

All these results provide very strong evidence that sMunc18-1 induces liposome clustering, a conclusion that was later confirmed by cryo-EM (see below). These data also show that the time dependence of the light scattering caused by liposome clustering (as the particle size approaches the wavelength of the light used in the experiments) is similar to that observed in the NBD fluorescence de-quenching assays (Fig. 1), and that much of the increase in light scattering (and increase in particle size) can be reversed by trypsinolysis of sMunc18-1, indicating that only a limited amount of membrane fusion or hemifusion occurs under the conditions of these experiments (estimated at ≤10%). Notably, sMunc18-1 did not induce liposome clustering in the presence of 1 M NaCl, perhaps because the high salt concentration hindered sMunc18-1/membrane interactions, but addition of 1 M NaCl did not reverse the liposome clustering observed after incubating sMunc18-1 with liposomes for 10 min (Table 1). These results suggest that an irreversible process underlies the liposome clustering activity of sMunc18-1, even if the clustering itself can be largely reversed by trypsinolysis.

### Liposome clustering induced by sMunc18-1 denaturation

**Figure 4:**
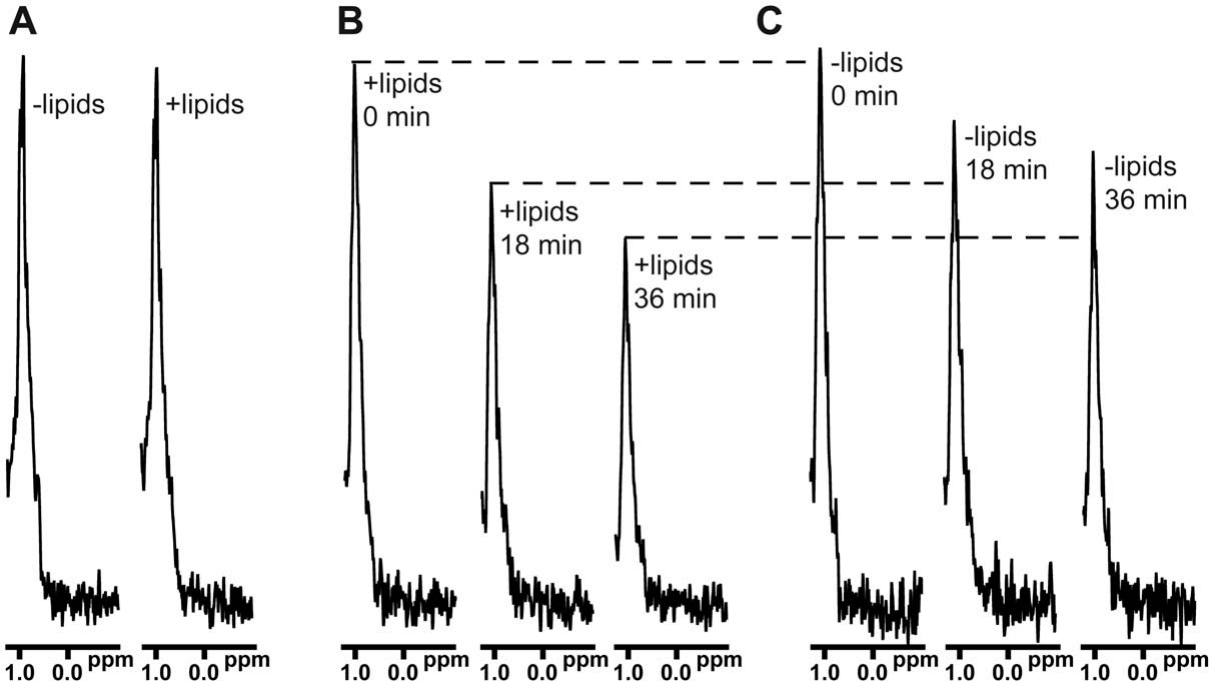
Time-dependent binding of sMunc18-1 to lipids. (**A**) 1D ^13^C-edited ^1^H-NMR spectra of 2 µM ^13^C-labeled sMunc18-1 in the absence or presence of liposomes (POPC∶DOPS 85∶15 molar ratio; 1 mM lipids) at 25°C. (**B**) 1D ^13^C-edited ^1^H-NMR spectra of the same sample containing liposomes in panel (**A**) acquired as a function of time after raising the temperature to 37°C. (**C**) 1D ^13^C-edited ^1^H-NMR spectra of the same sample lacking liposomes in panel (**A**) acquired as a function of time after raising the temperature to 37°C.

A natural mechanism of protein-induced liposome clustering entails the simultaneous binding of a protein to two membranes, as shown for synaptotagmin-1 [42], [43], the Ca^2+^ sensor for neurotransmitter release [44]. However, no direct evidence for Munc18-1 interactions with membranes has been reported, except for a small amount of binding to liposomes in co-floatation assays [45]. To test whether sMunc18-1 binds to membranes, we used a nuclear magnetic resonance (NMR) assay that monitors the intensity of the strongest methyl resonance (SMR) of a ^13^C-labeled protein in 1D ^13^C-edited ^1^H-NMR spectra [46]. In this assay, binding to an unlabeled protein or macromolecule is manifested by the decrease in the SMR intensity of the ^13^C-labeled protein associated with the increased effective molecular weight upon complex formation; in the case of binding to liposomes, the SMR of the ^13^C-labeled protein is broadened beyond detection because of the very large size of the liposomes (>100 MDa), as shown for synaptotagmin-1 [46]. However, we did not observe any significant decrease in the SMR intensity of ^13^C-labeled sMunc18-1 upon addition of liposomes (1 mM lipid concentration) at 25°C (Fig. 4A), showing that sMunc18-1 does not bind to the liposomes under these conditions.

Since the lipid mixing assays (Figs. 1A–D) and DLS experiments (Figs. 2,3) were performed at 37°C and the observed effects (increased NBD fluorescence intensity and particle size, respectively) were time dependent, we performed additional liposome binding assays at 37°C using the same NMR method. We did not observe any significant decrease in the SMR intensity of ^13^C-labeled sMunc18-1 in the presence of liposomes immediately after raising the temperature from 25°C to 37°C, but we did observe that the SMR intensity decreased over time (Fig. 4B). In control experiments performed in the absence of liposomes, the SMR intensity of ^13^C-labeled sMunc18-1 also decreased over time (Fig. 4C), but to a lesser extent than in the presence of liposomes. A natural explanation for these results is that sMunc18-1 is somewhat unstable at 37°C and partial denaturation leads to aggregation, resulting in a decreased SMR intensity. The stronger decreases of the SMR intensity in the presence of liposomes indicate that the denatured sMunc18-1 binds to the liposomes. These results suggest a mechanism for the liposome clustering activity of sMunc18-1 whereby denaturation of the protein exposes its hydrophobic residues, and insertion of distinct parts of the denatured protein into two different membranes helps to bring them together. It is also plausible that the liposome clustering may in turn help promoting sMunc18-1 denaturation, leading to a further decrease in the SMR intensity of sMunc18-1; however, if this is case, the destabilizing effect of the liposomes on sMunc18-1 cannot be very strong, since the liposomes caused only a moderate enhancement of the decrease in SMR intensity (Figs. 4B,C).

**Figure 5:**
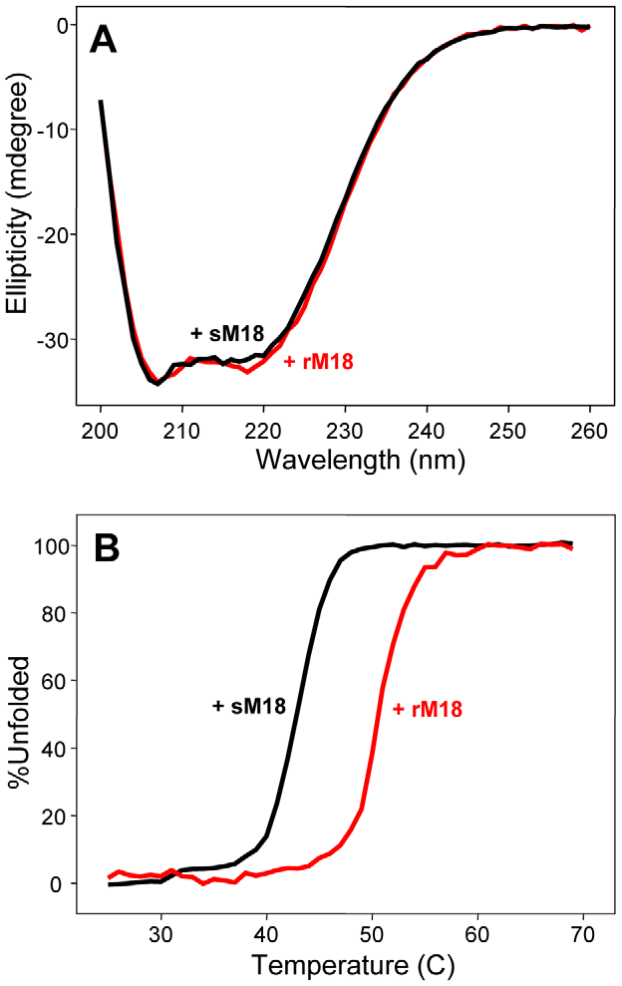
sMunc18-1 is less stable than rMunc18-1. (**A**) CD spectra of sMunc18-1 (black) and rMunc18-1 (red) at 25°C in phosphate buffer saline (PBS), pH 7.4. (**B**) Thermal denaturation curves of sMunc18-1 (black) and rMunc18-1 (red) monitored through the CD absorption at 220 nm. The T_m_ values calculated for sMunc18-1 and rMunc18-1 are 43°C and 51°C, respectively.

To investigate the stability of sMunc18-1, we used circular dichroism (CD) and thermal denaturation. The CD spectrum of sMunc18-1 is very similar to that of rMunc18-1 (Fig. 5A), as expected from the similarity of their three-dimensional structures [28], [47], and both proteins exhibit highly cooperative thermal denaturation curves characteristic of well-folded proteins (Fig. 5B). However, the mid point of the thermal denaturation curve (T_m_) of sMunc18-1 is 43°C, about 8°C lower than that of rMunc18-1. These data show that sMunc18-1 is only marginally stable at 37°C, supporting the notion that denaturation underlies its liposome clustering activity. Moreover, these results show that sMunc18-1 is markedly more unstable than rMunc18-1, which correlates with the observation that sMunc18-1 induces stronger increases in NBD fluorescence intensity than rMunc18-1 in the reconstitution assays (Fig. 1A).

**Figure 6:**
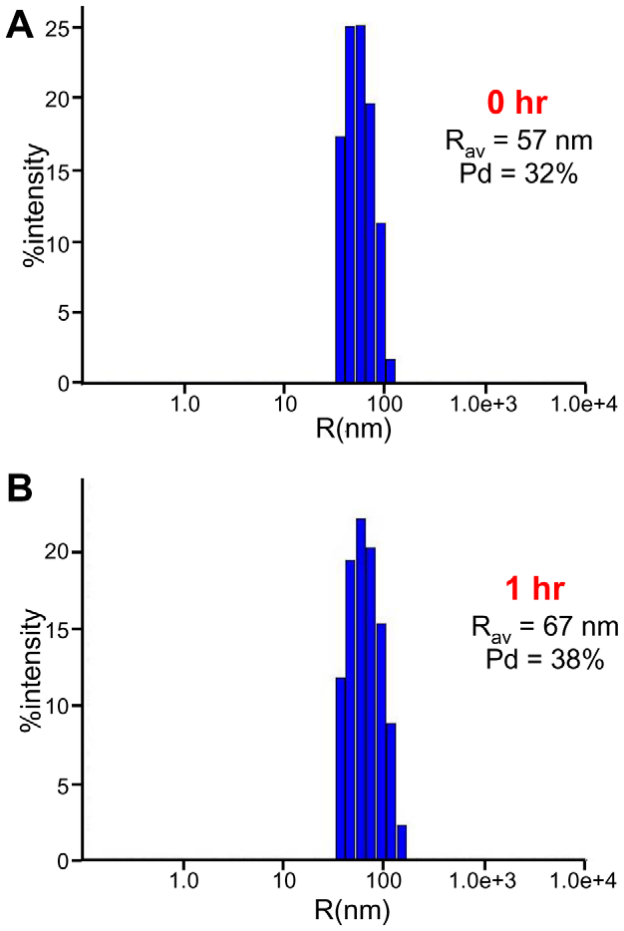
Glycerol hinders the liposome clustering activity of sMunc18-1. (**A,B**) DLS measurements of particle size in samples containing 15% glycerol, protein-free liposomes (POPC∶DOPS 85∶15 molar ratio; 30 µM lipids) and 4 µM sMunc18-1 right after mixing (**A**) and after 1 hr incubation at 37°C (**B**). The average radius (R_av_) and polydispersity (Pd) are indicated.

To further test whether the liposome clustering activity of sMunc18-1 arises from its instability, we examined whether this activity is altered by syntaxin-1, since syntaxin-1 binding to Munc18-1 is known to stabilize both proteins [29], [48]. Indeed, preincubation of sMunc18-1 with syntaxin-1 strongly impaired its liposome clustering activity, but addition of syntaxin-1 did not reverse the clustering induced by sMunc18-1 after a 10 min incubation with liposomes (Table 1), again suggesting that an irreversible process underlies the clustering activity, as suggested by the experiments with 1 M NaCl. Moreover, sMunc18-1 did not induce liposome clustering during 2 hr at 25°C and only a small degree of clustering after overnight incubation at this temperature (Table 1), in agreement with the notion that the clustering observed at 37°C arises from thermal denaturation of a fraction of sMunc18-1 molecules. Finally, since glycerol is a well-known protein-stabilizing agent, we tested whether addition of glycerol affects this ability. We found that sMunc18-1 did not induce liposome clustering in the presence of 15% glycerol, even after 1 hr incubation (Fig. 6). Altogether, these results provide very strong evidence that the liposome clustering activity of sMunc18-1 arises from denaturation.

### Visualization of sMunc18-1-induced liposome clustering and hemifusion by cryo-EM

**Figure 7:**
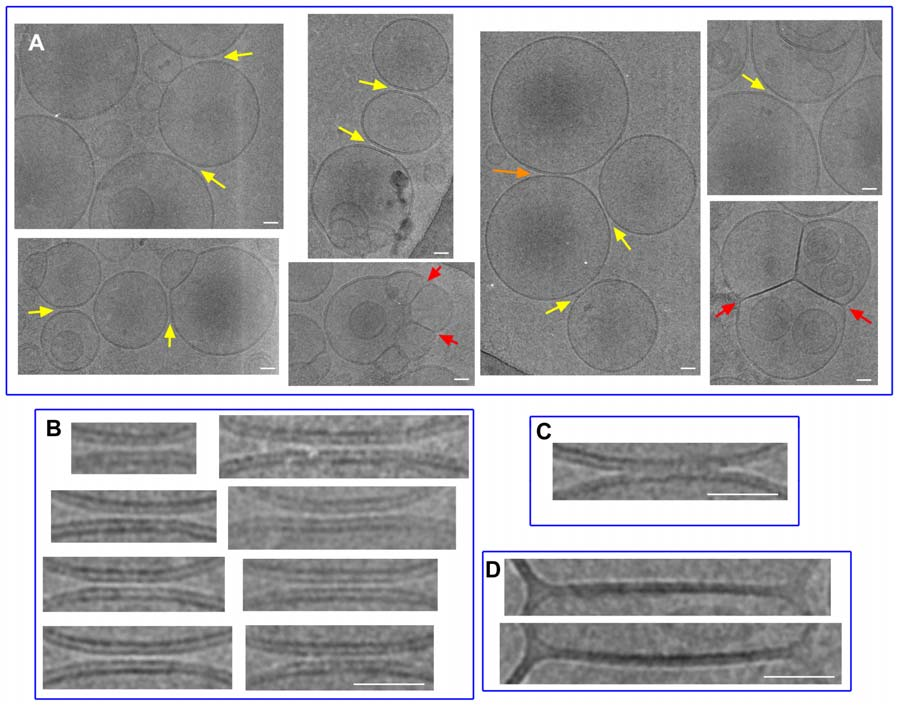
Cryo-EM analysis of liposome clusters induced by sMunc18-1. (**A**) Gallery of cryo-EM images of selected liposome clusters observed in a sample containing sMunc18-1 (30 µM) and liposomes (POPC∶DOPS 85∶15 molar ratio; 2.5 mM lipids). The sample was incubated for 5 min at 37°C after mixing, and was fast-frozen after loading onto the EM grid. (**B**–**D**) Expanded views of close interfaces between liposomes (**B**), of one interface where the bilayers appear to have been mixing at the moment the sample was frozen (**C**), and of hemifusion diaphragms (**D**). The three types of liposome interfaces are indicated with yellow, orange and red arrows, respectively, in panel (**A**). The scale bars correspond to 20 nm.

To better understand the effects of sMunc18-1 on membranes, we used cryo-EM. Control experiments in the absence of sMunc18-1 revealed dispersed vesicles with spherical shapes and the expected size, as observed before [23], [49]. In contrast, large liposome clusters were observed in the presence of sMunc18-1 (Fig. 7A), with abundant close interfaces between liposomes (Fig. 7A, yellow arrows; Fig. 7B). In most cases, the distances separating the two apposed bilayers were similar and small. We estimate that the intermembrane distances are 2 nm or less, and they are certainly smaller than the molecular dimensions of folded sMunc18-1 (ca. 4.5 nm×6 nm×8 nm) based on its crystal structure [47]. It is also noteworthy that the surfaces of the membrane-membrane interfaces were variable and in the larger surfaces there was clear membrane flattening (Fig. 7A, yellow arrows; Fig. 7B). In one case, the two membranes appeared to be in contact and the interface between them was blurry (Fig. 7A, orange arrow; Fig. 7C), suggesting that the two membranes were merging at the moment the sample was fast-frozen. We also observed multiple examples of clear hemifusion (Fig. 7A, red arrows; Fig. 7D).

**Figure 8:**
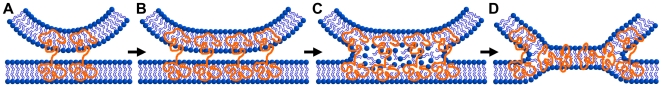
Proposed of model of how a denatured protein can induce membrane hemifusion without proceeding through a stalk intermediate. The model postulates that denatured proteins (represented as orange randomly shaped curves), and perhaps other amphpathic macromolecules, can induce hemifusion by binding to two membranes (**A**), accumulating at the membrane-membrane interface (**B**), and causing a scrambling of lipid molecules at the interface (**C**) that eventually rearranges into a stable hemifusion diaphragm (**D**). A curved membrane from a vesicle and a flat membrane are used in the drawings, but the mechanism could apply to membranes with diverse curvatures.

These observations suggest a model of protein-mediated membrane hemifusion that does not proceed through the stalk intermediate (Fig. 8). The close proximity observed in the membrane interfaces (Fig. 7A,B) suggests that sMunc18-1 must be inserted in both membranes to bridge them, most likely in a partially or totally denatured state that maximizes interactions of the hydrophobic sMunc18-1 side chains with the hydrophobic interior of the bilayers. It seems likely that multiple sMunc18-1 molecules bridge the two membranes, and that the denatured sMunc18-1 molecules bring the two membranes as close as possible to maximize interactions with both bilayers. The surface of the interface likely depends on the number of sMunc18-1 molecules bridging the membranes, and membrane flattening is necessary to keep the intermembrane distance constant in the larger interfaces containing more sMunc18-1 molecules. We speculate that destabilization of the two closely apposed bilayers by the denatured sMunc18-1 molecules may allow scrambling of the lipid molecules (Fig. 7C) to form a single bilayer, thus resulting in a hemifusion diaphragm (Figs. 7A,D; see model of Fig. 8). This latter event most likely involves a high energy barrier and occurs with low probability, since only limited amounts of lipid mixing (Fig. 1E) and hemifusion (Fig. 7A) are induced by sMunc18-1.

## Discussion

Reconstitution approaches provide powerful tools to understand the functions of the different components of intracellular membrane fusion machineries. However, because of their very in vitro nature, the validity of the results obtained with these approaches needs to be verified by establishing clear correlations with functional data obtained in vivo. Thus, initial reconstitution experiments led to the proposal that the neuronal SNAREs constitute a minimal membrane fusion machinery [16], but this model was in stark contrast with the critical dependence of synaptic vesicle fusion on additional factors in vivo, and very diverse results have been obtained in subsequent reconstitution experiments with neuronal SNAREs alone [3]. Particularly crucial is to understand the role of Munc18-1 and members of the SM protein family in general, and models for how these proteins may play a central role in fusion have been proposed [3], [9]. This overall notion has been supported by some reconstitution studies revealing stimulation of lipid mixing between SNARE proteoliposomes by Munc18-1 [31], [38], [40], or a strict requirement of Munc18-1 for lipid mixing [39]. However, it is still unclear whether Munc18-1 played an indirect role in these experiments by assisting in SNARE complex assembly, or had a direct role in membrane fusion. The study presented here now brings a new twist to this story by showing that sMunc18-1 can strongly perturb membranes by itself, although this activity appears to require denaturation. Our results also have implications for the interpretation of the widely used NBD fluorescence de-quenching assays, and suggest a novel mechanism of membrane hemifusion induced by amphipathic macromolecules.

An obvious question that arises from our finding that denatured sMunc18-1 can bridge membranes and induces hemifusion is whether this activity has any physiological relevance at all. The natural answer to this question is no, as it seems counterintuitive that denaturation underlies the specific function of Munc18-1 in a highly regulated process such as neurotransmitter release. Moreover, denaturation of multiple proteins unrelated to membrane traffic, such as α-lactalbumin, has previously been shown to induce liposome fusion ([50] and references cited therein), and just stabilizing sMunc18-1 with glycerol is sufficient to prevent its liposome clustering activity (Fig. 6).

Nevertheless, it is always advisable in science to keep an open mind, and the possibility that the membrane-perturbing activity of sMunc18-1 uncovered here might be somehow related to its biological function needs to be considered. In this context, it is worth noting that the fusogenic activities of diverse proteins mentioned above were normally observed at low pH values that also destabilize membranes [50]. In contrast, our results with sMunc18-1 were obtained at physiological pH. In addition, since a 10 min incubation causes denaturation of only a small fraction of sMunc18-1 (Fig. 4) but is sufficient to yield massive liposome clustering (Fig. 2, Table 1), it appears that sMunc18-1 is quite efficient in bridging membranes. Note also that we cannot rule out the possibility that membrane bridging by sMunc18-1 might not require denaturation but instead might involve an as yet unidentified conformational state of sMunc18-1 that may not be easily reached in the absence of an activator. Furthermore, some evidence has suggested that Sec1p, the SM protein involved in yeast exocytosis, has a function after SNARE complex formation [51]. Provocative models assigning a direct, central role for SM protein-membrane interactions in membrane fusion can be envisioned based on this observation, the known critical importance of SM proteins for membrane fusion in vivo [9], [25], [26], and the results reported here. For instance, SNARE complex assembly might bring the membranes into close proximity and attract the SM protein to the intermembrane space, where denaturation (or some form of activation) of the SM protein would lead to insertion into the two membranes to destabilize the bilayers and induce fusion. Clearly, these ideas must be considered highly speculative at this point, but they should be kept in mind in future research on the enigmatic function of Munc18-1 and SM proteins in general.

Regardless of whether the function of sMunc18-1 is in any way related to the membrane bridging activity described here, the cryo-EM images of Fig. 7 suggest an interesting mechanism of how amphipathic macromolecules can bridge membranes and induce hemifusion without proceeding through a stalk intermediate (Fig. 8). It is uncertain whether this mechanism can occur physiologically and whether some extension of this mechanism can lead to full membrane fusion in vitro or in vivo. Note for instance that the formation of extensive double-membrane diaphragms, as proposed in Figure 8, is very unlikely to occur in synaptic vesicle fusion because of the small size of synaptic vesicles and because it seems incompatible with the rapid formation of fusion pores to release neurotransmitters. Moreover, ample evidence has supported the notion that the stalk mechanism provides the most favorable pathway to merge two membranes from an energetic point of view [4]. However, it is still important for studies of membrane fusion to realize that mechanisms such as that illustrated in Fig. 8 might be plausible and hence must be considered when interpreting experimental results. Thus, insertion of disordered or partially disordered polypeptides into membranes is expected to strongly perturb the normal energy landscape that governs lipid-lipid and lipid-water interactions, which might enable alternative membrane-merging mechanisms that do not involve a stalk intermediate. It is also worth noting that homotypic vacuolar fusion involves the formation of a large, flat double-membrane ring that is excised at the vertex and remains in the organelle lumen after fusion [52]. While it is likely that such excision occurs through multiple fusion events around the vertex ring involving the canonical stalk mechanism, formation of the vertex ring provides a structural correlate between an intermediate in the fusion of biological membranes and the double-membrane diaphragms induced by sMunc18-1.

Our results also bring a note of caution for the methodology used to study membrane fusion in vitro. NBD fluorescence de-quenching experiments have become widely used and undoubtedly provide a powerful tool to study lipid mixing between membranes. However, there is a strong tendency in the literature to conclude that two membranes have fused based only on the observation of an increase in NBD fluorescence at a particular wavelength, despite the fundamental conceptual difference between lipid mixing and membrane fusion. This difference has been strongly emphasized by a recent study reporting that complementary DNA oligonucleotides incorporated into separate liposomes can induce 80% lipid mixing while yielding only 2% contents mixing [53]. Our data now show that increases in the fluorescence intensity at the wavelengths characteristic of NBD emission can arise from the light scattering associated with liposome clustering. Since a key step for proteins to induce lipid mixing and membrane fusion is to bring membranes together, such membrane bridging is expected to initially cause liposome clustering, at least to a certain extent that will depend on the relative rates of clustering and fusion. Hence, some degree of liposome clustering is likely to occur during lipid mixing assays, and at least part of the increases in NBD fluorescence observed may not reflect fluorescence de-quenching due to lipid mixing, but rather light scattering due to liposome clustering.

The relative contribution of light scattering to the increase in apparent NBD fluorescence intensity depends not only on the amount of liposome clustering but also on the specific parameters used to acquire the data, and can be estimated by recording full fluorescence emission spectra at the beginning an the end of a lipid mixing assay (e.g. Fig. 1E), or by examining the effects of trypsin (Fig. 3). Appropriate controls can also be used to correct for scattering, but it should be kept in mind that some typical controls commonly used in the study of SNARE function, such as addition of the cytoplasmic region of one of the SNAREs, may not account for scattering contributions because the reagent itself may inhibit liposome clustering. Our data show that the increase in apparent NBD fluorescence intensity due to scattering is not very large in absolute value even when large liposome clusters are formed (Figs. 1E, 2B). However, since the NBD fluorescence intensity at the beginning of a lipid-mixing assay is normally very small due to very efficient FRET to the Rho-labeled lipids, the contribution from scattering can yield a substantial increase in the apparent NBD fluorescence intensity in relative terms. For instance, F1/F0 was larger than 1.4 at the end of the experiment illustrated in Fig. 1C, blue circles, which was performed with only fluorescence-labeled v-SNARE liposomes and sMunc18-1, and hence could not reflect de-quenching due to lipid mixing. For comparison, values of F1/F0 between 1.5 and 1.6 would correspond to one round of fusion [43], based on a conversion method commonly used to quantify NBD fluorescence de-quenching assays [54], if all the fluorescence increase arose only from membrane fusion.

We would like to emphasize that the above observations do not imply that the conclusions of many published studies that relied primarily on NBD fluorescence de-quenching assays are necessarily wrong. However, our results provide compelling evidence that the simple observation of an increase in NBD fluorescence intensity in these assays is far from demonstrating membrane fusion, or even lipid mixing when the increase is moderate. To demonstrate membrane fusion, it is critical to demonstrate contents mixing without leakiness. In this context, it is worth noting that an increasing number of methods are being developed for this purpose, or to monitor lipid mixing through the development of FRET rather than fluorescence de-quenching (e.g. [17], [20], [40]). Nevertheless, the potential contribution of scattering due to liposome clustering needs to be considered for any assay using light in bulk solution. Moreover, our data also show that, as efforts to reconstitute intracellular membrane fusion machineries with an increasing number of components continue, it will become increasingly important to consider the potential effects of protein denaturation in the results obtained.

## Materials and Methods

### Protein expression and purification

DNA vectors to express the following proteins were described earlier: rMunc18-1 [27], sMunc18-1 (a kind gift from W. Weissenhorn) [47], rat synaptobrevin 2 [23] and t-SNARE complex formed by co-expression of human SNAP-25 and rat syntaxin-1A [45]. SNAP-25 had its four cysteines mutated to serine. The proteins were expressed in *Escherichia coli* BL21 (DE3) cells and purified as described [23], [30], [36], [45].

### Preparation of Liposomes and Reconstitution of the SNAREs

Two different lipid compositions were used for preparation of liposomes and proteoliposomes: i) 1-palmitoyl-2-oleoyl-sn-glycero-3-phosphatidylcholine (POPC) ∶1,2-dioleoyl-sn-glycero-3-phospho-L-serine (DOPS) 85∶15 (molar ratio), which is widely used in SNARE reconstitutions [16]; and ii) and POPC∶1-palmitoyl-2-oleoyl-sn-glycero-3-phosphoethanolamine (POPE) ∶DOPS∶phosphatidylinositol (PI) ∶cholesterol 50∶20∶10∶10∶10, which is similar the lipid composition of presynaptic membranes [55]. All lipids were obtained from Avanti Polar Lipids and kept in chloroform at -20°C. For fluorescent donor liposomes, 3% POPC was replaced with 1.5% N-NBD-1,2-dipalmitoyl-sn-glycero-3-phosphatidylethanolamine (NBD-PE) and 1.5% N-(lissamine rhodamine B sulfonyl)-1,2-dipalmitoyl-sn-glycero-3-phosphatidylethanolamine (Rho-PE). Protein free-liposomes were prepared by hydrating dried lipid mixtures with reconstitution buffer (25 mM HEPES pH 7.4, 100 mM KCl, 2 mM MgCl_2_, 2 mM DTT), vortexing thoroughly for 5 minutes, subjecting the sample to 5 freeze/thaw cycles, and extrusion through 80 nm pore size filters for at least 21 times. Final preparations contained 15 mM lipids. For reconstitutions of proteoliposomes with the so-called ‘direct’ method, SNARE proteins solubilized in reconstitution buffer containing 1% octyl-β-D-glucopyranoside (OG) were inserted into preformed liposomes and the detergent was removed by dialysis in the presence of SM2 Biobeads (Bio-Rad) as described [19], [23]. For reconstitutions by the ‘standard’ method, dried lipids and SNARE proteins where co-solubilized together in reconstitution buffer containing 1-2% OG, the suspensions were quickly diluted, the detergent was removed by dialysis, and the liposomes were isolated by floatation in a Histodenz gradient as described [16], [23], [31]. Stocks of the final proteoliposome preparations typically contained 5 mM lipids with the desired composition and 5 µM SNARE proteins.

### Lipid Mixing Assay

Lipid mixing assays were performed basically as described [23]. Briefly, aliquots of stock solutions of liposomes or proteoliposomes were mixed in reconstitution buffer at the desired final concentrations (see figure legends), and the NBD fluorescence at 533 nm was monitored as a function of time using a Photon Technology Incorporated (Lawrenceville, NJ) spectrofluorimeter (excitation at 460 nm). The reactions were performed at 37°C in 50 µl Quartz fluorometer cuvettes (Nova Biotech) preincubated at 37°C. For selected experiments (e.g. Fig. 1E), fluorescence emission scans were acquired at the beginning and after 1 hr of reaction. The same spectrofluorimeter was used to monitor the apparent fluorescence signal intensity at 375 nm as a function of time in the scattering assay of Figure 3C (350 nm excitation).

### Dynamic Light Scattering Tests

DLS experiments were performed on a Protein Solutions DynaPro instrument equipped with a temperature-controlled microsampler (Wyatt Technology), using 10 s acquisition time and 10% laser power. The samples were prepared in reconstitution buffer in a total volume of 20 µl, and each measurement was done as an average of 30 data points. The samples were normally diluted to a final lipid concentration of 30 µM lipids and centrifuged at 13,000 rpm for 10 min before data acquisition. For the kinetic experiments of Figures 3A,B, samples contained 100 µM lipids to mimic the conditions used in the lipid mixing assays. The results were processed with the program Dynamics V6. The radii and the size distribution were calculated with the regularization algorithm provided by this software.

### NMR spectroscopy

1D ^13^C-edited ^1^H NMR spectra were acquired on a Varian INOVA600 spectrometer equipped with a cold probe as described [46]. Samples contained 2 µM ^13^C-labeled sMunc18-1 dissolved in 20 mM HEPES (pH 7.4), 120 mM KCl, 1 mM TCEP, with or without liposomes (POPC∶DOPS 85∶15 molar ratio; 1 mM lipids), and 5% D_2_O. For each spectra, 1,000 scans were averaged (18 min total acquisition time).

### Circular dichroism

CD spectra were recorded on an Aviv model 62DS spectropolarimeter using a 1 mm path length cell with rMunc18-1 or sMunc18-1 samples dissolved in PBS buffer (pH 7.4). Thermal denaturation curves were monitored from the CD absorption at 220 nm. The fraction of unfolded protein at each temperature was calculated using the formula 100*(I_obs_−I_f_)/(I_u_−I_f_), where I_obs_ is the observed signal intensity, and I_u_ and I_f_ are the signal intensities of the unfolded and folded states, respectively.

### Cryo-electron microscopy

Quantifoil 200 mesh copper grids covered with a holey carbon film (R2/2, 2 µ round holes and 4 µ period) were glow-discharged in a Denton Vacuum DV-502A instrument with a 40 mA current for 45 s. A piece of Whatman filter paper with a droplet of 0.5 µl amylamine was used during glow discharge to render the carbon film partially hydrophobic in order to prevent extensive sticking of liposomes onto the film. To prepare the samples for cryo-EM, liposomes (POPC∶DOPS 85∶15; 2.5 mM lipids) were incubated with 30 µM sMunc18 in reconstitution buffer containing 1 mM MgCl_2_ at 37°C for 5 min. Control samples were prepared by an analogous procedure but without sMunc18-1. To make sure there were enough vesicles trapped in the holes of the grids after plunging freezing, a double-loading procedure was used. Aliquots of 3 µl were first loaded onto the carbon side of glow-discharged Quantifoil grids, incubated for 10 s and blotted with a piece of Whatman #4 paper from the edge of the grid for 5 s with a thin layer (∼0.5 microliter) solution left on the grid surface. Another aliquote of 3 µl of the sample was immediately loaded onto the same side of the grid. The grid was then loaded into a pre-conditioned Mark III Vitrobot, and was blotted 2.5 seconds before plunged into liquid ethane. The humidity in the Vitrobot chamber was kept above 90%, and a standard Vitrobot Filter paper (Ø55/20 mm, Grade 595) was used for blotting. The samples were kept in liquid nitrogen until EM imaging. To take images from a frozen grid, it was loaded into an Oxford cryoEM holder, and transferred into a JEOL 2200FS FEG transmission electron microscope equipped with an energy filter. The samples were kept at cryo temperatures and images were taken in a minimal-dose mode at 61.95K calibrated magnification, and recorded in a 2Kx2K Tietz slowscan CCD camera (with 1.69 post-column magnification) or on Kodak SO-163 films. The electron dose was kept at 20–30 electrons/Å^2^ during each exposure. A 35 eV energy filter was used for each exposure, and the defocus level was varied between −1.5 and −2.5 microns. Films were developed in full-strength D19 developer for 12 minutes, selected on an optical bench, and scanned in a flat-bed ZEISS SCAI scanner at 14 micron resolution. Due to massive vesicle clustering, an extensive examination of the entire area of each grid was necessary for samples containing sMunc18-1 in order to find the good and representative areas for imaging.
